# Preparation and Antibiofilm Properties of Zinc Oxide/Porous Anodic Alumina Composite Films

**DOI:** 10.1186/s11671-018-2568-4

**Published:** 2018-07-09

**Authors:** Shuying Xu, Tong Sun, Qian Xu, Changping Duan, Yue Dai, Lili Wang, Qiushi Song

**Affiliations:** 1 School of Metallurgy, Northeastern University, Shenyang, 110004 China; 2 College of Chemistry and Chemical Engineering, Bohai University, Jinzhou, 121013 Liaoning China; 3 College of Food Science and Project Engineering, Bohai University, Jinzhou, 121013 Liaoning China; 4 State Key Laboratory of Advanced Special Steel, School of Materials Science and Engineering, Shanghai University, Shanghai, 200072 China; 5 The 404 Company Limited, China Nation Nuclear Corporation, Lanzhou, 730000 China; 6 Liaoning Anjoy Food Co., Ltd., Anshan, 114100 Liaoning China; 7 Laboratory Management Center, Bohai University, Jinzhou, 121013 Liaoning China

**Keywords:** PAA/ZnO, Composite film, Antibiofilm, *Shewanella putrefaciens*

## Abstract

The PAA (porous anodic alumina) films were prepared by two-step anodic oxidation after different times, and then the ZnO/PAA composite films were prepared by sol-gel method on their surface. Meanwhile, the ZnO/PAA composite films were characterized by X-ray diffraction (XRD), thermogravimetric/differential thermal analyzer (TG/DTA), Fourier transform infrared spectrometer (FT-IR), scanning electron microscopy (SEM), transmission electron microscopy (TEM), selected area electron diffraction (SAED), and water contact angle (CA). The antibiofilm properties of ZnO/PAA composite films on *Shewanella putrefaciens* were measured simultaneously. The results show that the micromorphologies of PAA and ZnO/PAA composite films are affected by second anodization time. ZnO is a hexagonal wurtzite structure, and ZnO particles with a diameter of 10–30 nm attach to the inner or outer surfaces of PAA. After being modified by Si69, the ZnO films translate from hydrophilia to hydrophobicity. The ZnO/PAA film with the optimal antibiofilm properties is prepared on the PAA surface by two-step anodization for 40 min. The adherence of *Shewanella putrefaciens* is restrained by its super-hydrophobicity, and the growth of biofilm bacteria is inhibited by its abundant ZnO particles.

## Background

As we know, the bacteria can adhere to solid surfaces and form a slippery biofilm in appropriate environments [1]. Usually, the bacteria biofilms stick firmly to the surfaces of materials, such as stainless steel [2], rubber [3], glass [4], and polystyrene [5]. Biofilm would result in equipment corrosion [6] and food contamination [7], leading to huge economic losses. Many studies have indicated that biofilm adhesion is affected by the properties of material surface, such as roughness [8–11], microstructure [12, 13], hydrophilia [14–17], and antibiotic constituents [18–20]. Bohinc et al. [10] pointed out that the bacterial adherence would increase with the surface roughness of the glass. Singh et al. [12] demonstrated that high surface roughness can improve protein adsorption and accelerate the bacterial adhesion and biofilm formation. Bonsaglia et al. found that *Listeria monocytogenes* adhered to hydrophilic surfaces (e.g., stainless steel and glass) better than to hydrophobic ones (e.g., polystyrene) [14]. Other studies also proved that hydrophobic surface was not good for biofilm adhesion [16, 17]. Some studies have shown that antibiotic constituents could inhibit biofilm formation [18–20]. Three hundred four Cu-bearing stainless steel surfaces have great antibacterial and antibiofilm properties, taking advantage of the antimicrobial activity of Cu element [18]. In short, the surface properties are crucial to the antibiofilm properties of the materials.

Aluminum materials have been widely used, and porous anodic alumina (PAA) has drawn more attention in the fields of light electrical function, catalytic function, and sensing function in recent years [21–24], and its antimicrobial activity was reported. Ferraz et al. [24] reported that PAA can induce the adhering activation of monocytes/macrophages due to their matrix phase and nanoporosity.

In addition, zinc oxide (ZnO) thin films have been studied as an excellent material for antibacterial and antifungus. The adherence of *Pseudomonas aeruginosa* to ZnO films with nanorod surface structures was weaker than that of glass and sputtered ZnO, and more *P. aeruginosa* are killed in the ZnO films [25]. Meanwhile, one research pointed out that ZnO-coated surfaces dramatically restricted biofilm formation, and the generation of hydroxyl radicals played a key role in antibiofilm activity but not the existence of zinc ions [26]. Furthermore, ZnO composite films can be used in many fields to restrict biofilm formation and will have good application prospects in aquatic product preservation [27]. ZnO is hydrophilic, while hydrophobic films are good at restraining biofilm adhesion. Thus, it is necessary to improve the hydrophobic properties of ZnO film.

Aquatic products are very perishable due to their microbial spoilage [28]. Under aerobic storage conditions, *Pseudomonas* spp. and *Shewanella putrefaciens* are known as dominant spoilage organisms [29]. *Shewanella putrefaciens* has psychrotrophic nature and can reduce trimethylamine-*N*-oxide to trimethylamine [30]. So, *Shewanella putrefaciens* will be used as the indicator bacteria in this paper.

The microstructures of ZnO films would be different due to their PAA base, and then the antibiofilm properties would be affected. In this work, ZnO films were prepared on PAA with different morphology and modified to improve the hydrophobicity. The antibiofilm properties of *Shewanella putrefaciens* of the ZnO/PAA composite films were studied. The results provide potential value for the applications in food packaging, food processing equipment, and the other antibacterial functional material fields.

## Materials and Methods

### Materials

All the reagents used in this study were analytically pure. The de-ionized and sterile water was used to prepare solutions with conductivity lower than 0.5 mS/cm. *Shewanella putrefaciens* ATCC8071 was purchased from American Type Culture Collection. Aluminum foils of 0.3 mm thickness with aluminum purity over 99.99% were purchased from Shengshida Metal materials Co., Ltd. (China).

### Preparation of ZnO/PAA Composite Films

#### Preparation of Porous Anodic Alumina (PAA) Films

A high purity aluminum foil was cut into small dimension of 10 × 30 mm^2^ and was polished with polishing paste of 50 nm silica by a polisher (WV80, Positec Machinery Co., Ltd., China) and was ultrasonic degreased in acetone at 53 kHz, 280 W for 15 min (SK8210HP, Kudos Ultrasonic Instruments Co. Ltd., Shanghai). Then, the foils were washed two times both with ethanol and water, respectively. The pretreated aluminum foils were used as the anode, the equal-area graphite sheet as the cathode, and the 0.3 mol/L oxalic acid solution as the electrolyte. The first anodization was under the conditions of 30 °C and 40 V for 90 min. After that, the aluminum sheets were immersed in the mixed solution of 6.0 wt% H_3_PO_4_ and 1.8 wt% H_2_CrO_4_ at 60 °C for 4 h to remove the alumina layers. The second anodization was then performed under the same conditions but for 0, 40, 60, and 80 min, respectively. The porous anodic alumina (PAA) films with a different port model were obtained.

#### Preparation of ZnO/PAA Composite Films

Firstly, the equal volume of 0.02 mol/L zinc acetate ethanol solution and 0.04 mol/L NaOH ethanol solution were mixed under rapid stirring at 70 °C for 5 min, and then the PAA films (aluminum foils) were immersed in the mixed solution under a vacuum degree of − 0.085 MPa. Afterwards, the solution was heated to boiling. After it became thin blue sol, the aluminum foils were taken out and rinsed with de-ionized water. Then, the samples were vacuum dried at − 0.085 MPa, 80 °C for 6 h, and the ZnO/PAA composite films were prepared after calcined at 480 °C for 2 h in air atmosphere. The zinc oxide powders were prepared simultaneously. Finally, the ZnO/PAA composite films and the powders were modified by 1.0 wt% Si69 ethanol solution at 65 °C for 2 h and then vacuum dried at − 0.085 MPa, 40 °C for 12 h.

### Characterization of ZnO/PAA Composite Films

X-ray diffraction of the zinc oxide powders was performed using X-ray powder diffractometer (Rigaku Ultima IV, Rigaku, Japan) at a step of 0.02°and 2*θ* range of 10°–80° with CuKa radiation of 40 kV, 50 mA. The thermal changes and weight loss of the samples were analyzed by thermogravimetric/differential thermal analyzer (TG/DTA, Perkin Elmer Diamond). Fourier transform infrared (FT-IR) spectra were recorded with a Scimitar 2000 Near FT-IR Spectrometer (Agilent, American) in the range of 4000–400 cm^−1^. The surface micrographs of PAA films and ZnO/PAA composite films were imaged by field emission scanning electron microscopy (FESEM, S-4800, Hitachi, Japan). The nanoparticle morphologies shaved from the ZnO/PAA composite films are measured by field emission transmission electron microscopy (FETEM, Jem-2100F, JEOL, Japan), and the selected area electron diffraction (SAED, Jem-2100F, EOL, Japan) of the samples were examined. The water contact angles (CA) of the composite films (before/after modified) were measured by the sessile drop method at several different positions on each sample surface using 3.0-μL droplets of de-ionized water (SL200B, USA).

### The Antibiofilm Properties of ZnO/PAA Composite Films

#### Cultivation of *Shewanella putrefaciens* Biofilm

The bacterium suspension of secondary activating *Shewanella putrefaciens* (OD_595_ ≈ 0.5) and alkaline peptone water (APW) of 3% (m/v) NaCl were mixed uniformity by the ratio of 1:200 (*v*/*v*). ZnO/PAA composite films (0.5 × 0.5 cm) were immersed in the diluted inoculums of 3 mL and incubated at 28 °C for a certain time. Under this condition, *Shewanella putrefaciens* grew well and showed strong proliferative ability.

#### Adhesion Assay of *Shewanella putrefaciens* Biofilms on ZnO/PAA Composite Films

After cultivating in bacterium suspension of *Shewanella putrefaciens* for a certain time, the ZnO/PAA composite films with biofilm were transferred to another sterile centrifuge tubes and washed three times with 1 mL of 0.85% (m/v) sterile NaCl solution to remove the free bacteria. The biofilm was stained with 1 mL of 0.2%*w/w* crystal violet for 15 min at room temperature and was washed three times with 1 mL of 0.85% (m/v) sterile NaCl solution to remove redundant crystal violet. Then, the stained biofilms were ultrasonically stripped in 33% (*v*/*v*) acetic acid of 200 μL at 53 kHz, 280 W for 10 min. The OD_595_ (optical density at 595 nm) of the above solution was recorded by a VICTOR™ X3 microplate reader (Perkin Elmer, America) in the 96-well microtiter plates. The results were shown as “averages ± standard deviations” of the thrice parallel experiment.

#### Total Bacterial Count Assay of *Shewanella putrefaciens* Biofilm on ZnO/PAA Composite Films

The ZnO/PAA composite films with biofilm were washed three times with sterile phosphate-buffered saline (PBS, pH 7.4; 137 mmol/L NaCl, 2.7 mmol/L KCl, 10 mmol/L Na_2_HPO_4_, and 1.8 mmol/L KH_2_PO_4_) to dislodge floating bacteria, and the stained biofilms were ultrasonically stripped in 10-mL sterile PBS at 53 KHz, 280 W for 10 min. Subsequently, the total bacterial count in the biofilms was measured by plate count method. With the thrice parallel experiment, the results were shown as “averages ± standard deviations,” and the colony growth curve of the biofilm bacteria was drawn.

#### The Micrographs Measurement of *Shewanella putrefaciens* Biofilms

After removal of the floating bacteria, the ZnO/PAA composite films with biofilm were immersed in 2.5% (*w*/*v*) glutaraldehyde at 4 °C for 4 h. Subsequently, the samples were dehydrated every 30 min with 50, 70, 80, and 90% (*v*/*v*) ethanol, respectively. After being dipped in the absolute ethyl alcohol for 1 h, the samples were naturally air dried on a clean bench. The surface micrographs of the samples were imaged by FESEM (S-4800, Hitachi, Japan) after gold sputter coated at 3 kV for 40 s.

#### The CLSM Measurement of *Shewanella putrefaciens* Biofilms

The ZnO/PAA composite films with biofilm were washed with phosphate-buffered saline (PBS, pH = 7.4) for three times to remove the floating bacteria, and the samples were stained in the dark for 15 min in the mixed solution of 0.01wt% acridine orange (AO, Sigma, America) and 0.1wt% propidium iodide (PI, Sigma, America). After that, the samples were washed three times with PBS to dislodge the redundant dyeing solution, and the excessive moisture was dislodged. Ten microliters of anti-fluorescence quenching sealing agents (Biosharp BL701A, China) was dropped on the biofilms, and the samples were stored at 4 °C without light. The proportions of alive and dead cells of the biofilms were observed using confocal laser scanning microscope (CLSM, TCS-SP5 II, Germany Leica Instrument Co., Ltd.) [31, 32].

## Results and Discussion

### Characterization of ZnO Films

#### XRD Characterization of the ZnO Powders Prepared by Sol-gel Process

**Fig. 1:**
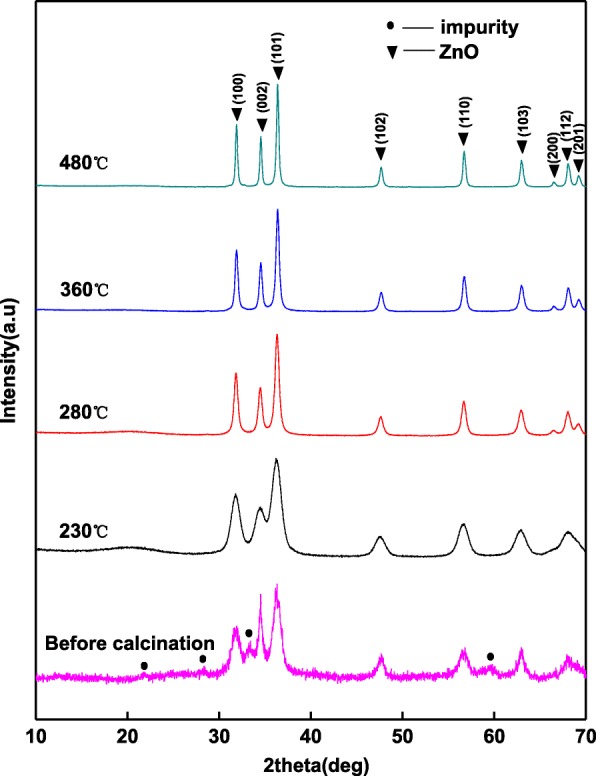
XRD patterns of the zinc oxide powders calcined at different temperatures

The antibacterial and antibiofilm properties of zinc oxide are affected by its crystal structure [33, 34]. Figure 1 shows that the crystal structure of the samples is transformed after calcined. Before calcinations, the samples are contained in the hexagonal wurtzite structure of ZnO. The diffraction peaks at 31.70°, 34.52°, 36.31°, 47.68°, 56.82°, 62.92°, and 67.92° of 2*θ* correspond to (100), (002), (101), (102), (110), (103), and (112) crystal planes of zinc oxide (PDF # 36-1451, *a* = *b* = 3.250 and *c* = 5.207), respectively. The wide diffraction peaks indicate low crystallinity and small particles of ZnO. Meanwhile, less impure peaks reveal the intermediate in the sample. After calcined at 230 °C, the impure peaks disappear and the measured noise decreases, but the width of the diffraction peaks is invariable. It means that the intermediate disappears, and the degree of the crystal increases. With the increasing of calcining temperature, the diffraction peaks of ZnO become sharpen, indicating that the crystallinity increases and the crystal particles grow.

Only bound water from Zn(CH_3_COO)_2_·2H_2_O is produced in the ethanol solution of Zn(CH_3_COO)_2_, and the hydrolyzation of CH_3_COO^−^ is inhibited. Firstly, the Zn(CH_3_COO)_2_ is hydrolyzed and produced the intermediate product.

4Zn(CH_3_COO)_2_·2H_2_O → Zn_4_O(CH_3_COO)_6_ + 2CH_3_COOH + 3H_2_O(1)

In the heating process, the collosol is facilitated by the ethanol solution of NaOH, and the space steric effect of CH_3_COO^−^ is of great importance for the stability of ZnO collosol. Meanwhile, the neutral reaction of CH_3_COOH with NaOH happens.

5Zn_4_O(CH_3_COO)_6_ + 22NaOH + 13H_2_O → 4Zn_5_(OH)_8_(CH_3_COO)_2_·2H_2_O + 22CH_3_COONa(2)

CH_3_COOH + NaOH→CH_3_COONa + H_2_O(3)

Spanhel and Anderson [35] indicated that the zinc oxide alcogels are formed from ZnO grains through aggregation and Ostwald Growth (aging). Then, the intermediate of Zn_5_(OH)_8_(CH_3_COO)_2_·2H_2_O is heated and decomposed into ZnO phase [36, 37]. Thus, the hexagonal wurtzite structure of ZnO is the basis of the dried gelatin before calcinations.

Zn_5_(OH)_8_(CH_3_COO)_2_·2H_2_O → 5ZnO + 2CH_3_COOH + 5H_2_O(4)

Hosono et al. [37] have confirm this reaction mechanism. The ethanol solution of Zn(CH_3_COO)_2_·2H_2_O turned colloidal products during heating at 60 °C, and XRD results show that the dry product of gelatin is a mixture of crystalline ZnO and Zn5(OH)_8_(CH_3_COO)_2_·2H_2_O. After refluxing for 48 h, the particles are transformed into the wurtzite ZnO.

#### TG/DTA Analysis

**Fig. 2:**
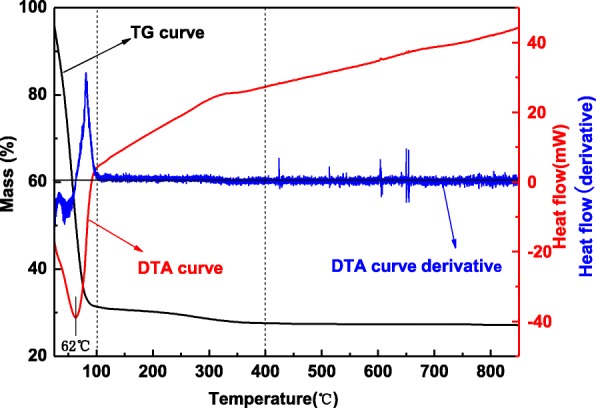
TG/DTA graphs for zinc oxide gelatin

The TG/DTA result of zinc oxide gelatin is shown in Fig. 2, and the TG curve could be divided into three stages. In the first stage, the mass loss is 68.6% from room temperature to 100 °C, and an endothermic peak existed at 62 °C. It is corresponding to the lost ethanol solvent and water in zinc oxide gelatin. In the second stage, the mass loss is only 3.8% from 100 to 400 °C. XRD results show the impurity disappeared, the increased crystallinity, and the growth of crystal particles after calcined at 230, 280, and 360 °C, respectively. A small amount of mass loss may be the loss of pore water and the transition of the impurity. From 400 to 850 °C, there is no mass loss and endothermic peak, indicating no crystal transformation in this stage. Meanwhile, the XRD result shows the crystal grows after calcination at 480 °C. The results of TG/DTA are consistent with the XRD results.

#### FT-IR Characterization of the Unmodified/Modified Zinc Oxide Films

**Fig. 3:**
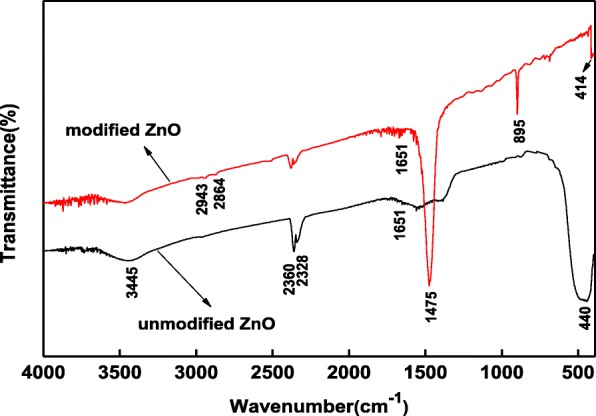
FT-IR spectra of the unmodified/modified zinc oxide films

Figure 3 shows the FT-IR spectra of the unmodified and hydrophobic modified ZnO films. The wide peaks at 3600–3300 cm^−1^ are attributed to the stretching vibration of −OH, and the peak at 1651 cm^−1^ is attributed to the bending vibration of –OH, respectively, indicating the absorbed water and capillary water in the samples [38]. The peaks at 2360 and 2328 cm^−1^ are attributed to the carbon dioxide in the air. The peaks at 2943 and 2864 cm^−1^ are due to asymmetric and symmetric stretching vibrations of −CH_2_, respectively. The stronger peak at 1475 cm^−1^ is ascribed to the in-plane bending vibration or scissoring vibration of −CH_2_ groups [39], and the peak at 895 cm^−1^ is ascribed to the stretching vibrations of Si-O groups [40]. The peaks at about 440 and 414 cm^−1^ are ascribed to the framework vibration of Zn-O groups of the unmodified/modified ZnO [41]. The results indicate that modification makes –S–S– bonds of Si69 rupture, and triethoxysilylpropyl is grafted on the samples, so the hydrophobic properties of ZnO films increase. Wang [42] reported that nano-ZnO dispersion could be improved by in situ modification of Si69 and Si69 grafted on the surface of nano-ZnO particles through the chemical reaction. This is consistent with our analysis results.

#### Micromorphologies Analysis of PAA Films

**Fig. 4:**
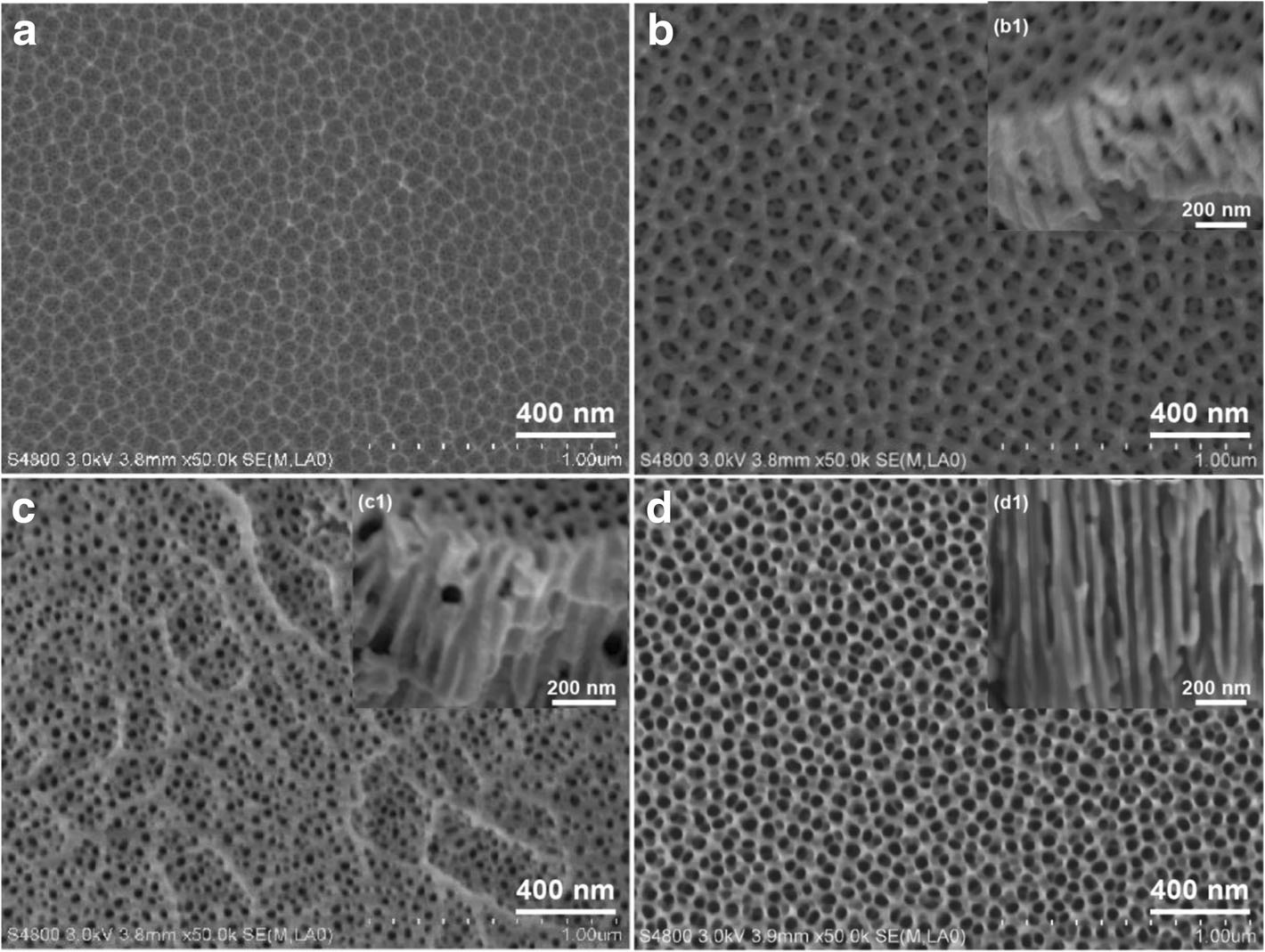
SEM images of the porous anodic alumina (PAA) with different times of second anodization duration **a** 0 min, **b** 40 min, **c** 60 min, and **d** 80 min

The morphologies of PAA films are affected by the second anodized oxidation time. As shown in Fig. 4, after removing the alumina layers of the first anodization, the PAA film is a serried hexagonal honeycomb frame with nanopores of 5–10 nm (Fig. 4a). After two-step anodization for 40 min, the nanopores are transformed into multilayer shell frames (Fig. 4b). After two-step anodization for 60 min, the multilayer shell frames fade away, and the nanopores’ diameter is enlarged to 20–40 nm, meanwhile there are the ridges on the surface (Fig. 4c). Extending the two-step anodization time to 80 min, the nanopores are enlarged to 60–70 nm, and the ridges are disappeared (Fig. 4d).

According to the theory of acidic field-assisted dissolution (AFAD) [43], in the anodization process, the oxide films of barrier layer became non-uniform, and the ridges are formed. At these points, the formation and development of the microporous are promoted by the aggravated AFAD. With the second anodization time prolonging, the ordered and through holes are formed gradually on the surface, and then the multilayer shell frames and ridges disappeared (Fig. 4b–d). The result is similar to Reddy’s, which prepared PAA via two-step anodization process in 0.3 mol/L oxalic acid [44].

#### Micromorphologies Analysis of ZnO Films

**Fig. 5:**
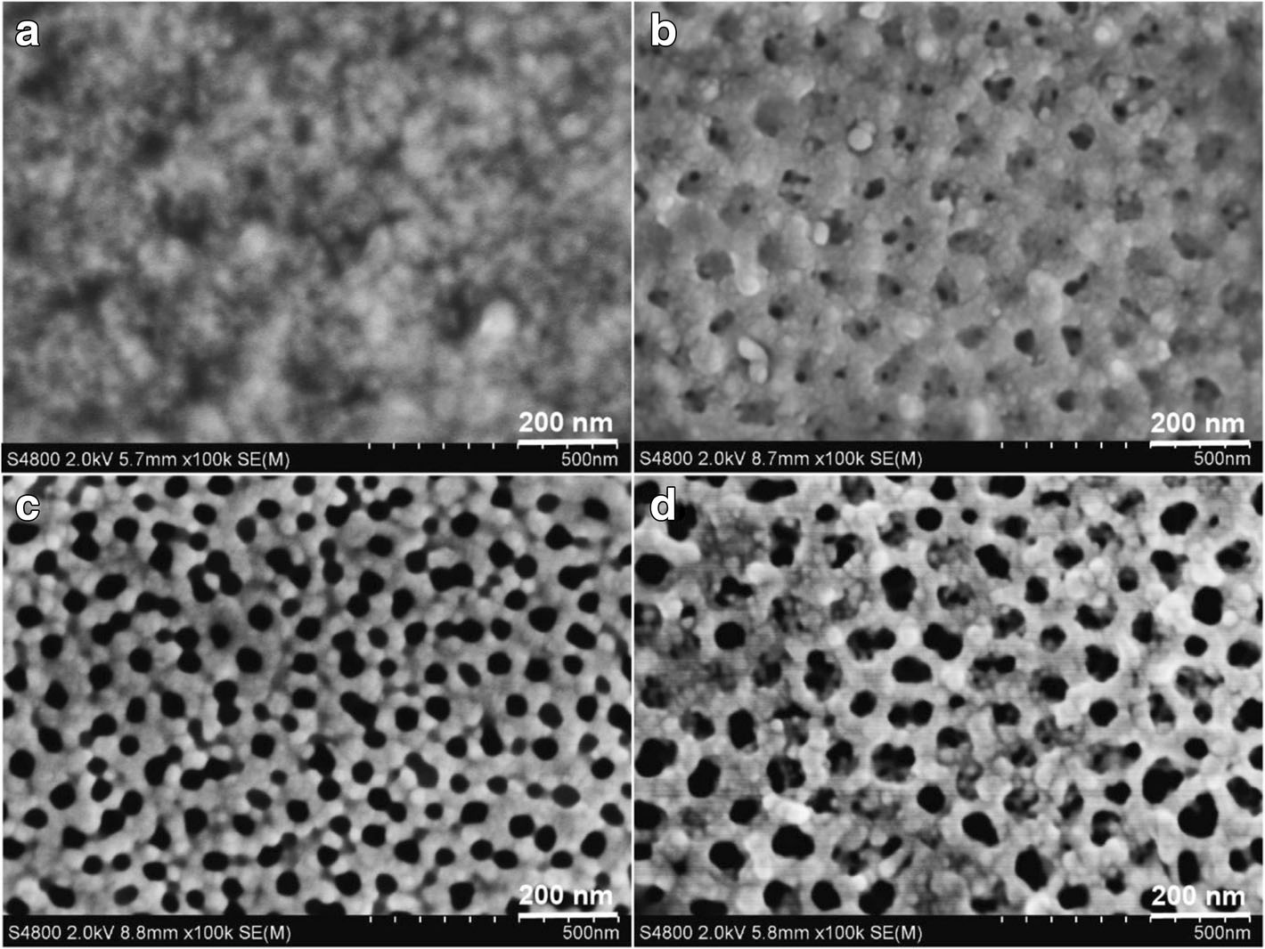
SEM images of the ZnO films prepared on PAA with different time of two-step anodization duration **a** 0 min, **b** 40 min, **c** 60 min, and **d** 80 min

The antobiofilm properties of the material surfaces are affected by their morphology and substances [12]. As shown in Fig. 5, the morphologies of ZnO films are significantly different which are prepared on the PAA films with different time of second anodization duration. On the surfaces of PAA film with nanopores of 5–10 nm, the agglomerated large particles of 20–30 nm are densely attached and formed thick ZnO films (Fig. 5a). On the surface of PAA film which is prepared by two-step anodization duration for 40 min, the multilayer shell frames are remained on the ZnO film (Fig. 5b). As shown in Fig. 5c, the ZnO particles have been attached to the skeleton of PAA film and formed larger holes. On the sample with nanopores of 60–70 nm, the ZnO particles of 10–20 nm are attached at the edge of the PAA holes, and a part of the particles entered the nanopores (Fig. 5d). This may be the collosol entering the larger holes under vacuum condition and then form the ZnO particles. The above results indicate that the smaller nanopore diameters of PAA, the higher sticking rate of ZnO is. Wu et al. [45] consider that the collosol particles are formed easily on the wall of the holes due to the negative of sol particles and the positive of PAA pore walls. The finding is also consistent with the study by Bousslama et al. [46]. The collosol just attaches at the wall of holes when the PAA film is immersed in zinc sol for 24 h, and then the holes are full for 48 h, indicating that the collosol particles attached preferentially at the wall of holes.

The above results show that the collosol particles easily enter into big holes and attach to the inner surface under vacuum condition; however, the collosol particles only attach to the skeletons of exterior surfaces of PAA with the small holes.

**Fig. 6:**
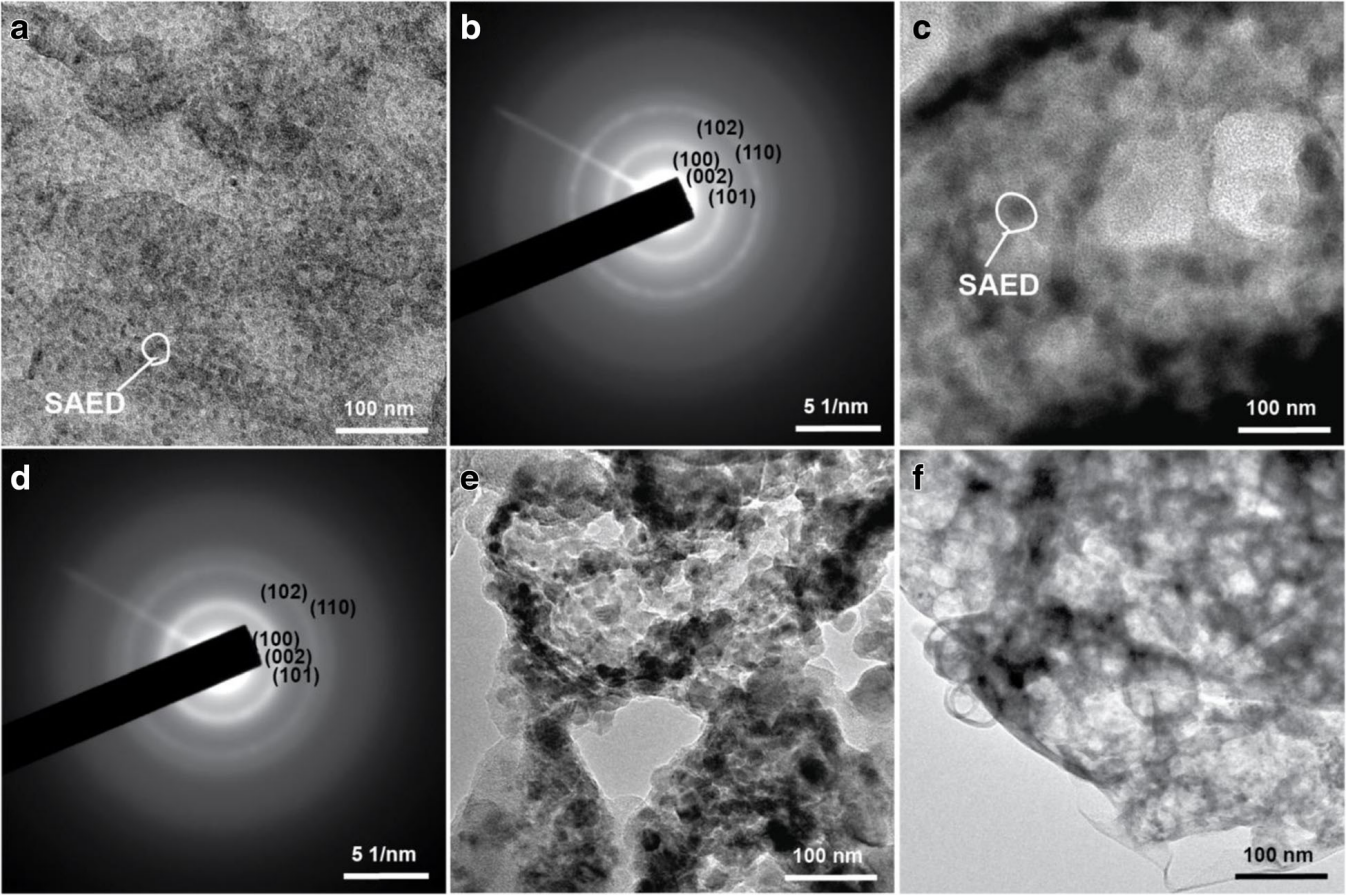
TEM images (**a**, **c**, **e**, **f**) and SAED patterns (**b**, **d**) of the ZnO films prepared on PAA with different time of two-step anodization duration **a**, **b** 0 min **c**, **d** 40 min; **e** 60 min; and **f** 80 min

The TEM images of the ZnO film shaved from the ZnO/PAA composite film are shown in Fig. 6. On the PAA surface prepared only by one-step anodization, the delaminated ZnO particles are about 10 nm, but the particles of 20–30 nm are shown in SEM image (Fig. 5a), indicating that the ZnO particles are agglomerated. On the PAA surface prepared by two-step anodization, the delaminated ZnO particles are approximately 20 nm, and a part of particles are agglomerated at the individual locations. It is manifested that the ZnO particles are attached at the edge of the PAA holes first (Fig. 6c, e, f).

The lattice planes (100), (101), (102), (110), and (103) of hexagonal wurtzite structure ZnO are shown in SAED patterns (Fig. 6b, d), indicating that ZnO is a hexagonal wurtzite. The results are coincident with the XRD analysis.

#### Hydrophobicity-Hydrophilicity Characterization of the ZnO Film Surface

To decrease the bacterial adhesion of the materials, the prepared ZnO films with different micromorphology are treated to improve the hydrophobicity, and the water contact angle of the thin film surface before and after modification is shown in Table 1.

**Table 1:** Water contact angle of the ZnO films prepared on PAA with different times of two-step anodization duration

Time of two-step anodization duration (min)	CA of the surface	
	Before modification (°)	After modification (°)
0	75.5 ± 0.3	125.5 ± 0.3
40	29.6 ± 0.3	149.8 ± 0.3
60	45.9 ± 0.3	107.1 ± 0.3
80	61.5 ± 0.3	99.3 ± 0.3

Before modification, the ZnO films are hydrophilic due to the surface hydroxyl groups on the ZnO particles. The hydrophilicity is the best due to its porous structure which prepared on PAA surface with two-step anodization duration of 40 min. For the other samples with two-step anodization duration of 60 and 80 min, the hydrophilies decreases gradually because of the low adhesive quantity of ZnO. For the sample with one-step anodization duration, the low hydrophily is due to its non-porous structure.

After modification, the ZnO films are translated into hydrophobic. According to FT-IR analysis, the triethoxysilylpropyl is grafted on the samples after –S–S– bonds of Si69 rupture. Meanwhile, it could be a result of its porous structure and more ZnO particles; the film has the highest hydrophobicity with two-step anodization duration of 40 min.

### Characterization of *Shewanella putrefaciens* Biofilms

Chi et al. [47] reported that anodized aluminum has no antibacterial activity to Gram-negative bacteria(*Escherichia coli* and *P. aeruginosa*) and Gram-positive bacteria (*Streptococcus faecalis* and *Staphylococcus aureus*). However, ZnO has an excellent antibacterial and antibiofilm activity [25–27], and there is a positive correlation between antibacterial and antibiofilm activity [48, 49]. Furthermore, the antibacterial properties of ZnO are affected by its microstructure [50, 51]. In order to obtain an excellent antibiofilm activity surface, the ZnO films with different microstructure were prepared on PAA films with different times of second anodization duration, and the antibiofilm properties were measured.

#### The Adhesion of the Biofilms and Growth Curves of the Biofilm Bacteria

The formation and development of bacterial biofilm can be concluded in five stages: the reversible adhesion of bacteria to the surface initially; the conversion from the reversible adhesion to the irreversible adhesion; the initial formation of the biofilm; the development of the matured biofilm; and the degenerating of the biofilm and the bacteria return to planktonic state [52].

**Fig. 7:**
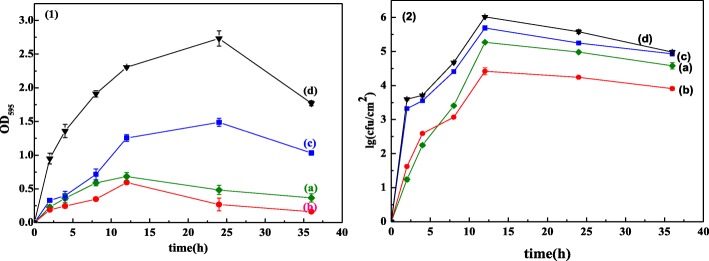
The adhesion of *Shewanella putrefaciens* biofilm (**1**) and colony growth curve of the biofilm bacteria (**2**) on the ZnO films prepared on PAA with different time of two-step anodization duration (a) 0 min, (b) 40 min, (c) 60 min, and (d) 80 min

As shown in Fig. 7(1), in 2 hours, the adherence of *Shewanella putrefaciens* biofilm on the ZnO films increases rapidly, illustrating the conversion from the reversible adhesion of bacteria to irreversible adhesion. From 2 to 12 h, the adherence of the biofilm gradually increases, which is the growth stage of biofilm. From 12 to 24 h, the adherence of the biofilm increases or declines slightly, manifesting the mature stage of biofilm. After 24 h, the adherence of biofilm declines, and the biofilms enter into the degenerating stage. Figure 7(2) shows that the variation tendency of the biofilm bacteria accords with the adherence of the biofilm, indicating that the development of the biofilm depends on the biofilm bacteria.

In addition, for the ZnO film prepared on the PAA surface with two-step anodization duration for 80 min, the adherence of the biofilm and the total amount of biofilm bacteria are both the highest among the four samples. However, for the ZnO film prepared on the PAA surface with two-step anodization duration for 40 min, the antibiofilm property is optimal. This could be because of the biofilm adherence that is inhibited by the highest hydrophobicity, and then less exopolysaccharides (EPS) and the other nutrient against the growth of biofilm bacterial. For the ZnO film prepared on the PAA surface with two-step anodization duration for 80 min, its hydrophily is good for the initial adherence of the biofilm, and less ZnO particles do not inhibit the growth of the biofilm bacteria. Meanwhile, more biofilm adhesive materials nourish the biofilm bacteria, and the biofilm bacteria multiply rapidly. Consistent with our research, the biofilm adherence is inhibited by the higher hydrophobicity of ZnO film in the initial stage of the biofilm formation [49]. The adherence of biofilms is affected by the hydrophobic and hydrophilic properties of the materials [14, 53, 54]. Bonsaglia et al. [14] reported that *L. monocytogenes* adhere to the hydrophilic surface more easily than to the hydrophobic surface. Many studies found that the bacterial adhesion is reduced or inhibited by hydrophobic surface [47, 54]. Shaer et al. [54] indicated that the biofilm colonization on the functionalizing orthopedic hardware could be prevented by hydrophobic polycations. Chen et al. [55] also suggested that the biofilm could be inhibited by low surface free energy. The results matched those from us.

#### The Morphological Characteristics of *Shewanella putrefaciens* Biofilm

**Fig. 8:**
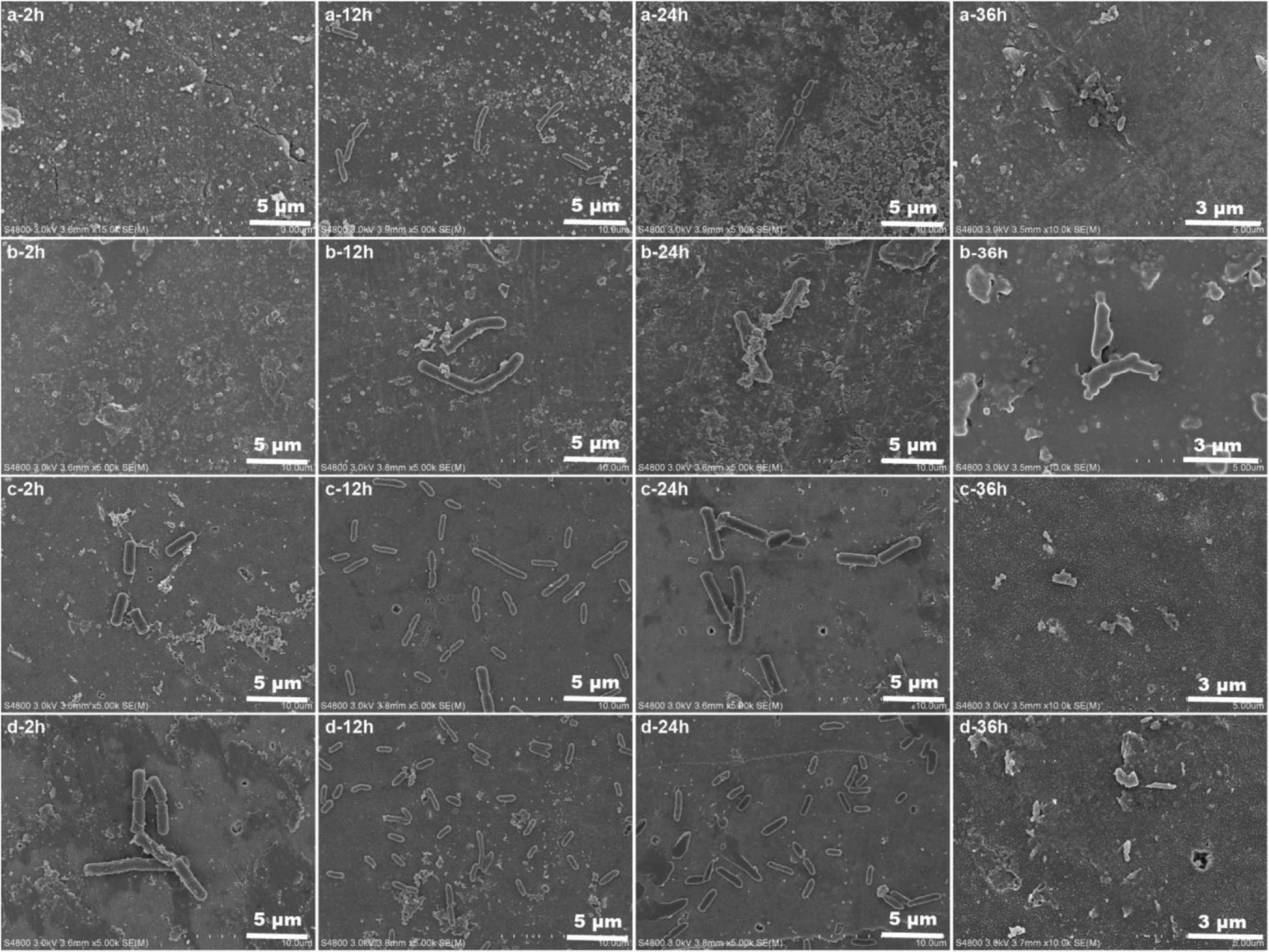
SEM images of the biofilms on the zinc oxide films prepared on PAA with different time of two-step anodization duration **a** 0 min, **b** 40 min, **c** 60 min, and **d** 80 min

The microtopographies of *Shewanella putrefaciens* biofilm at various stages are shown in Fig. 8.

After cultivated for 2 h, there are less adhesive materials on the ZnO film prepared on PAA without two-step anodization (a) and with two-step anodization duration for 40 min (b), but more adhesive materials and a few bacteria on the other two (c, d). It is indicated that the anti-adhesive properties of the former two are better than the latter two, it is consistent with Fig. 7. After cultivated for 12 h, more and more EPS and bacteria are attached to ZnO films, signifying the rapid growth of the biofilm. At 24 h, the EPS films are thickened gradually and biofilm bacteria grew well, indicating mature biofilms. At 36 h, the deciduous EPS films and dead bacteria illustrate the biofilm degenerating stage.

According to the antibacterial mechanisms of dissolved metallic ions, the dissolved zinc ions are combined with active proteinase of bacteria, make proteinase lose its bioactivity, and damage its bacterial cells to death [34, 56]. Thus, the antibacterial properties of the former two (a, b) are superior to the latter two (c, d) due to their plentiful ZnO particles on the films. Xie [57] and Jones [58] also thought that the antibacterial abilities strengthened with the dosage increasing of ZnO particles. Meanwhile, the adhesive materials and bacteria on the sample (d) are all more than the others, according to the analysis of adhesion of *Shewanella putrefaciens* biofilm and colony growth curve of the biofilm bacteria (Fig. 7). Feng et al. [59] found that the hypha of *Escherichia coli* easily reached into the PAA pores with diameters of 50 and 100 nm, and the biofilm accumulated and adhered to the surface of PAA. However, there is no hypha of *Shewanella putrefaciens* could be observed in our study. It can be inferred that the optimal antibiofilm properties are ascribed to the lower hydrophobicity of ZnO film in the initial stage of the biofilm formation.

#### The CLSM Characteristics of the ZnO/PAA Composite Biofilms

**Fig. 9:**
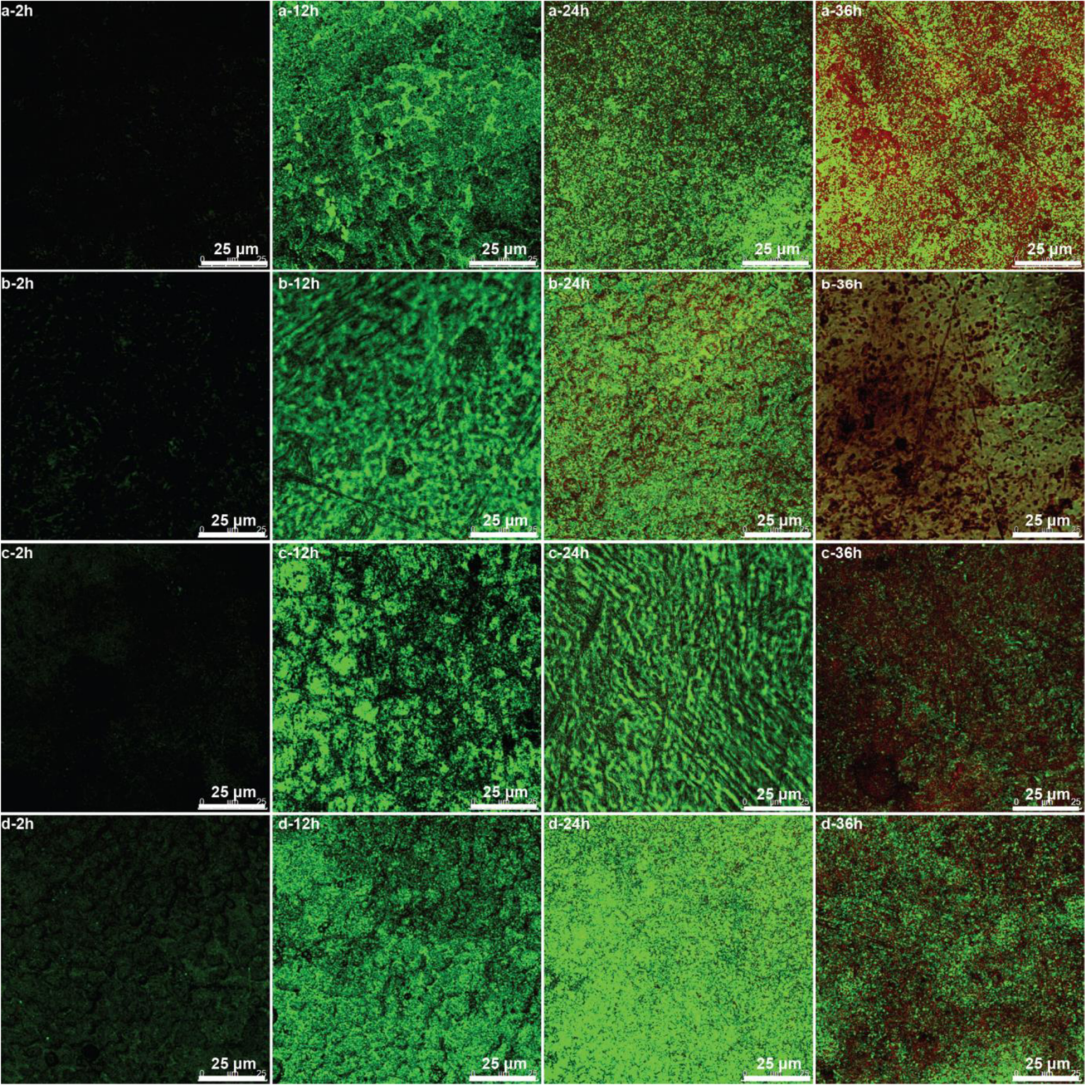
CLSM images biofilms formed on the zinc oxide films prepared on PAA with different time of two-step anodization duration **a** 0 min, **b** 40 min, **c** 60 min, and **d** 80 min

As shown in CLSM images, the live *Shewanella putrefaciens* bacteria are green, and the dead ones are red (Fig. 9). The black images indicate that the counts of live bacteria on the surfaces are few after biofilm cultivation for 2 h. Biofilm bacteria multiply rapidly, and the counts of live bacteria are significantly increased with the cultivation time. More dead bacteria are observed in the former two (a, b) at 24 h and in all samples at 36 h. The counts of dead bacteria of the latter two (c, d) are less than that of the former two (a, b). The results indicate that the antibacterial properties of the former two (a, b) are superior to that of the latter two (c, d), which is according to the previous analysis.

## Conclusions

In this work, the PAA films with different microstructures were prepared by two-step anodic oxidation first, and then the ZnO/PAA composite films are prepared by sol-gel. The ZnO films are hydrophilic due to the surface hydroxyl group on the ZnO particles. After being modified by Si69, the ZnO films translate to hydrophobicity because of its hydrophobic group. The antibiofilm properties of the ZnO films are affected by the hydrophobicity and amount of ZnO particles. The hydrophobicity inhibits the initial adherence of the biofilm and less EPS and the other nutrient against the growth of biofilm bacteria. So, the antibiofilm properties of the ZnO/PAA film are optimal which are prepared on the PAA surface with two-step anodization duration for 40 min because of its super-hydrophobicity and plenty of ZnO particles.

## Abbreviations

AO: Acridine orange
CA: Water contact angle
EPS: Exopolysaccharides
FT-IR: Fourier transform infrared spectrometer
PAA: Porous anodic alumina
PBS: Phosphate-buffered saline
PI: Propidium iodide
SAED: Selected area electron diffraction
SEM: Scanning electron microscopy
TEM: Transmission electron microscopy
TG/DTA: Thermogravimetric/differential thermal analyze
XRD: X-ray diffusion
